# A cross-sectional study on alcohol and contraception use among sexually active women of childbearing age: Implications for preventing alcohol-exposed pregnancies

**DOI:** 10.1177/17455057231161479

**Published:** 2023-03-31

**Authors:** Sherly Parackal, Mathew Parackal, Sumera Saeed Akhtar

**Affiliations:** 1 Centre for International Health, University of Otago, Dunedin, New Zealand; 2 Department of Marketing, University of Otago, Dunedin, New Zealand

**Keywords:** alcohol-exposed pregnancy, binge drinking, effective contraception use, New Zealand, periconceptional drinking

## Abstract

**Background:**

A high proportion of unwanted or unplanned pregnancies may be alcohol-exposed due to contraception failure or non-use. Nevertheless, data on contraception and alcohol use in the context of the risk of alcohol-exposed pregnancies are sparse.

**Objectives:**

To describe contraception use and alcohol consumption in sexually active non-pregnant women and investigate the factors associated with less effective contraception methods.

**Study Design:**

A cross-sectional national survey of women aged 18–35 years.

**Methods:**

Data from non-pregnant women who were sexually active (*n* = 517) were analysed. Descriptive statistics were used to report demographics, consumption, and contraception measures. Logistic regression was used to investigate the factors associated with less effective contraception among drinkers.

**Results:**

The majority of participants were younger (46%), of NZ European ethnicity (78%), not in a permanent relationship (54%), with some or completed tertiary education (79%), employed (81%) and not users of the community services card (82%). Twenty-five percent of women were smokers, 94% consumed alcohol, and 72% binged at least ‘monthly or less’. Most women used the pill (56%), and 20% of drinking women were using a contraception method with a 10% or more annual failure rate after 1 year of use. Women who binged ‘weekly or more often’ had similar odds of using less effective contraception as women who ‘never’ binged (*p* > 0.05). Younger Māori or Pacific women (odds ratio = 5.99; 95% confidence interval of odds 1.15*–*31.2; *p* = 0.033) and women who had no tertiary education (odds ratio = 1.75; 95% confidence interval of odds 0.00*–*3.06; *p* = 0.052) had higher odds of using less effective contraception.

**Conclusion:**

With 20% of women at risk of an alcohol-exposed pregnancy, public health measures to address alcohol consumption and the effective use of contraception are critical to reducing the risk for alcohol-exposed pregnancies in NZ.

## Introduction

Alcohol-exposed pregnancies can lead to lifelong disabilities in the offspring, a condition encapsulated in the umbrella term, foetal alcohol spectrum disorders (FASDs).^1^ The majority of women who consume alcohol in pregnancy do so prior to realizing they are pregnant, continuing their pre-pregnancy drinking behaviour through the early stages or the periconceptional period of pregnancy.^2,3^ This is concerning as the periconception period, that is, 1 month prior to conception and 2 months into the pregnancy,^4–6^ is extremely important in teratology research, as it represents the period of organogenesis when the foetus is most vulnerable to toxic exposures.^1,3,7,8^ The foetal consequences of maternal periconceptional drinking have been documented in the literature since the late 1980s, which includes spontaneous abortion,^4,9^ lowered apgar scores,^7^ elevated prevalence of congenital heart defects,^10^ omphalocele^11^ birth defects typical of the upper end of the FASD spectrum such as smooth philtrum and thin vermillion border^5,12,13^ craniofacial anomalies,^5^ increased risk of cleft lip with or without cleft palate,^5,12,14^ neural tube defects (NTDs),^14^ minor physical anomalies, and neurobehavioral deficits in older aged children.^1,4,5,15^

Periconceptional drinking prevalence ranges from 45% in the United States,^16^ 50% in New Zealand^2^ to around 80% in Ireland.^17^ Heavy/binge drinking during this period is also not uncommon. Pre-pregnancy binge drinking is a strong predictor for drinking as well as binge drinking in early pregnancy.^2,16^ Binge drinking in the periconceptional period is estimated to be 17% in New Zealand,^2^ 20% in Australia,^18^ 25% in Denmark,^19^ and 13% in Canada.^20^

In the US, unintended pregnancies, either due to contraception failure or sex without contraception accounted for 80% of pregnancies unknowingly exposed to alcohol.^21^ Pre-conception binge drinking has also been shown to be associated with unintended pregnancies^22^ and is a contributing factor for unprotected sex both in a university student population^23^ as well as the general population.^24^ Estimates from the 2002 Behavioural Risk Factor Surveillance System in the US indicate that 7.6% of women of childbearing age who were sexually active with a male partner were not using contraception, more than half consumed alcohol and about 12.4% were binge drinkers.^25^ Among women not intending a pregnancy but who were having unprotected sex with a male partner, 37.7% (95% confidence interval (CI) 32.1%–43.7%) were drinking alcohol.^26^

Despite the higher level of risk for an alcohol-exposed pregnancy among women of childbearing age, very few studies have investigated associations between alcohol consumption and contraception use in the context of increased risk of an alcohol-exposed pregnancy. Published studies that have investigated this association are more in the context of sexually transmitted diseases such as HIV^27^ and STIs,^28^ sexual risk behaviours such as multiple partners,^29^ and substance use.^30^ An intervention designed to address both risky drinking and effective contraception use was found to reduce the risk of an alcohol-exposed pregnancy among college students^31^ and in the community.^32^ To replicate such studies in countries where the risk for an alcohol-exposed pregnancy is high, an improved understanding of contraception use and drinking patterns in the context of alcohol-exposed pregnancies is imperative. New Zealand is one such country; however, data on the prevalence of contraception use and drinking patterns in the context of alcohol-exposed pregnancies are currently absent. The current study aimed to (1) describe contraception use and alcohol consumption in sexually active non-pregnant women and (2) investigate the factors associated with less effective contraception use to inform targeted public health initiatives to reduce alcohol-exposed pregnancies and hence the prevalence of FASD.

## Materials and methods

### Study design

The current study reports the findings of the analysis of data from a sub-set of women (*n* = 517) who participated in the 2015 *Periconceptional Alcohol Consumption (PAC) study* (*n* = 1062). These women reported their maternal status as ‘not pregnant’ and were sexually active with a male partner in the year preceding the survey. A detailed methodology of the study findings in relation to alcohol consumption prior to recognizing pregnancy has been published elsewhere.^33^ The study was a national cross-sectional hybrid survey^34^ of women in their peak childbearing years (18 to 35 years) in 2015. The study received ethics approval from the University of Otago Human Ethics Committee on 25 November 2015 (Ref 15/154). The STROBE cross-sectional reporting guidelines were used in reporting the study findings.^35^

### Sample size calculation and participant selection

Using simple random sampling 3250 names and addresses of female participants who met the age criteria (18–35 years, both ages inclusive) from the New Zealand Electoral Roll in December 2015 were contacted in anticipation of a 35%–50% response rate.

### Data collection

Data collection was achieved via a pre-tested questionnaire specifically designed to meet the objectives of the study.

1. Maternal status: responses to the question that asked participants’ current maternal status was used as a skip question to direct them to different sets of questions. Women who indicated that they were not currently pregnant, nor had a baby in the past 3 years nor were currently planning a pregnancy (not pregnant; *n* = 710) were directed to questions on sexual behaviour, contraception use, past year alcohol consumption, and other knowledge questions not reported here. Among the ‘not pregnant’ women, 517 were sexually active with a male partner and data from these women were used to achieve the objectives of this study.
2. Demographic measures: age data were collected using the ‘year of birth’ question, and any missing data were replaced by the age recorded on the electoral roll. Data on ethnicity, level of education, household income, employment status, and marital status were collected using standardized questions from the New Zealand 2013 Census (Statistics NZ, 2013b). From the ethnicity data, prioritized ethnicity was determined in the order of Māori > Pacific > Asian > New Zealand European/Other (NZEO). Due to inadequate numbers in the Pacific ethnic group, Māori and Pacific were combined to form one category Māori/Pacific. The level of education data was re-coded as ‘No tertiary education’ and ‘some or completed tertiary education’. Annual household income categories were collapsed to create four categories, namely ‘Less than 30,000’, ‘30,000 to 70,000’, ‘More than 70,000’, and ‘Prefer not to answer or don’t know’. Data were also collected on whether participants used a community service card, an indicator of socio-economic deprivation using yes/no options which were re-coded as 1 and 0, respectively. Due to a high proportion of missing or ‘don’t want to answer’ data points for the household income variable, the use of a community service card, a surrogate measure of deprived socioeconomic status in New Zealand^36^ was used in all the analyses. Data on marital status was collapsed to form two categories, namely, ‘In a permanent relationship’ and ‘Not in a permanent relationship’.
3. Consumption measures: frequency of consuming alcohol in the 12 months preceding the survey, number of standard drinks consumed on a typical drinking day, and frequency of consuming six or more standard drinks on one occasion (binged) at least ‘monthly or less’ were collected. The New Zealand standard drink definition was provided both in the written form as well as in graphic form to enable participants to provide data on the number of standard drinks consumed. This definition read as:
  A 330ml bottle/stubby or can of normal strength beer or a 30ml measure of spirits mixed or straight, or 1 can of ready-to-drink (RTD) contains around one standard drink. 100mls of wine is one standard drink, so a small 150mls glass of wine contains one and a half standard drinks, a medium 200mls wine glass contains two standard drinks and a typical 750ml bottle of wine contains around eight standard drinks.
  Data on smoking status was collected for the past year by asking ALL participants whether they smoked or not (never smoked and smoked) in the 12 months preceding the survey. Those who identified themselves as smokers were coded as ‘1’ and non-smokers as ‘0’.
4. Contraception use: information on the contraception method ‘usually’ used in the past 12 months was collected using a list of currently available contraception methods. For women who used more than one type of contraceptive, a prioritization framework developed by the Centers for Disease Control and Prevention^36^ was applied in the order of decreasing effectiveness: implant, male sterilization, IUS, IUD, injections, the pill, male condom, rhythm/temperature/calendar, diaphragm, female condom, withdrawal, foam and emergency contraception. These contraceptives were collapsed into two categories based on the probability of failure after 1 year of use^37,38^ as Very Effective (<10% annual failure rate and Less Effective (⩾10% annual failure rate).^21^

### Statistical analysis

All statistical analyses were conducted using SPSS VS 24 (IBM Corp, Armonk, NY, USA) with two-sided *p* < 0.05 considered statistically significant. Descriptive statistics were used to report the demographic makeup, consumption measures, and contraception measures. Logistic regression was used to investigate the factors associated with less effective contraception among drinkers. The outcome variable of interest was contraception failure after one year of use. The predictor variables of interest were smoking, frequency of binging, age, prioritized ethnicity, highest level of education, marital status, employment status, and use of a community service card as a surrogate for income level. Data missing for any variables of interest resulted in the removal of the case from the analysis.

## Results

The study achieved a response rate of 37%. The majority of sexually active non-pregnant women were younger in age (46%; 41.9–50.5), of NZ European ethnicity (78%; 74.1–81.3), not in a permanent relationship (54%; 50.0–58.7), with some or completed tertiary education (79%; 75.1–82.2), employed (82%; 78.2–85.0), and not users of the community services card (82%; 78.6–85.3). A quarter of these women were tobacco smokers (25%; 20.8–28.2), 94% (92.2–96.2) consumed alcohol, 37% (32.8–41.2) consumed alcohol 2–4 times a month, 80% (58.6–67.2) did not conform to the NZ guideline for responsible drinking and 76% (73.8–81.2) consumed six or more standard drinks on one occasion at least ‘monthly or less’ (Table 1).

**Table 1.** Demographic and substance use characteristics of sexually active non-pregnant women (*n* = 517).

Characteristic	% (95% CI; *n*)
Age category (years)	
18–24	46.2 (41.9–50.5; 239)
25–29	31.3 (27.3–35.3;162)
30–34	22.4 (18.8–26.0;116)
Prioritized ethnicity^a^	
Māori	14.3 (11.3–17.3;73)
Pacific	2.2 (0.9–3.4; 11)
Asian	5.9 (3.8–7.9; 30)
New Zealand European/ other	77.7 (74.1–81.3; 397)
Marital status^b^	
In a permanent relationship	45.7 (41.3–50.0; 231)
Not in a permanent relationship	54.3 (50.0–58.7; 275)
Highest level of education^b^	
Some secondary education or less	4.9 (3.1–6.8; 25)
Completed secondary education	16.4 (13.2–19.6; 83)
Some or completed tertiary education	78.7 (75.1–82.2; 398)
Current employment^b^	
Employed	81.6 (78.2–85.0; 413)
Unemployed	18.4 (15.0–21.8; 93)
Use of community service card^c^	
Yes	18.1 (14.7–21.4; 91)
Smoking^d^	
Yes	24.5 (20.8–28.2; 126)
Frequency of alcohol consumption in the past year^e^	
Never	5.8 (3.8–7.8; 30)
Monthly or less	28.1 (24.2–32.0; 145)
2–4 times a month	37.0 (32.8–41.2; 191)
2–3 times a week	22.7 (19.1–26.3; 117)
4–5 times a week	3.9 (2.2–5.5; 20)
6 or more times a week	2.5 (1.2–3.9; 13)
Typical day alcohol consumption^f^	
2 standard drinks or less	20.4 (32.8–41.4; 178)
More than 2 standard drinks	79.6 (58.6–67.2; 302)
Consume 6 or more standard drinks on one occasion(binge)^g^	
Yes	76.4 (73.8–81.2; 372)

CI: confidence interval.

a Missing data *n* = 6.

b Missing data *n* = 11.

c Missing data *n* = 13.

d Missing data *n* = 2.

e Missing data *n* = 1.

f Missing data *n* = 7.

g Excludes non-drinkers.

With regard to contraception use, the majority of women used the pill (56%; 51.4–60.0) and 21% (17.7–24.8) of women were using a contraception method with 10% or more annual failure rate after 1 year of use (Table 2).

**Table 2.** ‘Usual’ use of contraception (prioritized) in the year preceding the survey (*n* = 517).

Prioritized contraception type	% (95% CI; *n*)
Hormonal implant	15.7 (12.5–18.8; 81)
Male sterilization	2.9 (1.5–4.3; 15)
Depo-provera injections	4.4 (2.7–6.2; 23)
Birth control pill	55.7 (51.4–60.0; 288)
Male condom	16.2 (13.1–19.4; 84)
Rhythm/temperature/calendar/withdrawal/none^a^	5.0 (3.1–6.9; 26)
Contraception failure after 1 year of use Less effective (⩾10% annual failure rate)	
All	21 (17.7–24.8; 110)
Among drinkers	20.8 (17.2–24.4)

CI: confidence interval.

a *n* = 25.

As shown in Table 3, non-European women (*p* < 0.05) and women with no tertiary education (*p* = 0.057) were more likely to use contraception methods that had a 10% or more annual failure rate after 1 year of use. Women who binged ‘monthly or less’ were more likely to use effective contraception (*p* < 0.001)

**Table 3.** Demographics and substance use of sexually active non-pregnant women according to contraception failure (*n* = 517).

	<10% Annual failure rate	⩾10% Annual failure rate	Statistics
	% (95% CI; *n*)		
Demographic characteristics			
Age category (years)			
18–24	47.4 (42.6–52.3; 193)	41.8 (32.6–51.0; 46)	χ^2^ = 2.31 df = 3 *p* = 0.00
25–29	29.7 (25.3–34.2; 121)	31.5(28.2–46.3; 41)	
30–34	22.9 (18.8–26.9; 93)	20.9(13.3–28.5; 23)	
Prioritized ethnicity^a^			
Māori	13.6 (10.63–16.57; 55)	16.7(13.47–19.93; 18)	χ^2^ = 21.39 df = 2 *p* = 0.002
Pacific	1.0 (0.14–1.86)	6.5 (4.36–8.64)	
Asian	4.5 (2.4–6.5; 18)	11.1(5.2-17.0; 12)	
NZ European/other	80.9 (77.1–84.7; 326)	65.7 (56.8–74.7; 71)	
Marital status^b^			
In a permanent relationship	178 (44.6)	53 (49.5)	χ^2^ = 0.82 df = 1 *p* = 0.364
Not in a permanent relationship	221 (54.4)	54 (50.5)	
Highest level of education^b^			
No tertiary education	19.5 (15.7–23.4; 78)	28 (19.5–36.5; 30)	χ^2^ = 3.62 df = 1 *p* = 0.057
Some or completed tertiary education	80.5 (76.6–84.3; 321)	72 (63.5–80.5; 77)	
Current employment^b^			
Employed	82.2 (78.9–86.3; 328)	87.3 (71.8–87.1; 85)	χ^2^ = 0.43 df = 1 *p* = 0.512
unemployed	17.8 (14.1–21.7; 71)	19.7 (12.9–28.2; 22)	
Use of community service card^c^			
Yes	17.9 (14.1–21.7; 71)	19.0 (11.3–26.1; 20)	χ^2^ = 0.37 df = 1 *p* = 0.847
No	82.1; 78.3–85.9; 326)	81.0 (73.9–88.7; 87)	
Substance use			
Smoking^d^			
Yes	74.3 (70.1–78.6; 301)	73.6 (65.4–81.9; 81)	χ^2^ = 0.21 df = 1 *p* = 0.884
No	25.7 (21.4–29.9; 104)	26.4 (18.1–34.6; 29)	
Frequency of alcohol consumption in the past year^e^			
Never	5.2 (3.0–7.3; 21)	6.4 (3.1–13.3; 9)	χ^2^ = 6.41 df = 3 *p* = 0.094
Less than monthly	26.1(21.8–30.4; 106)	30.9 (26.5–44.4; 39)	
2–4 times a month	37.9 (33.2–42.7; 154)	40.7(24.8–42.5; 37)	
2 or more times a week	30.8 (26.3–35.3; 125)	22.7(14.9–30.6; 25)	
Typical day alcohol consumption^f^			
2 standard drinks or less	39.6 (34.8–44.4; 160)	44.8 (37.4–56.2; 51)	χ^2^ = 1.83 df = 1 *p* = 0.176
More than 2 standard drinks	60.4 (55.6–65.2; 244)	64.2 (43.8–62.6; 58)	
Frequency of consuming six or more standard drinks on one occasion (binged)^g^			
Never	20.3 (16.2–24.3; 78)	35.6 (26.3–45.0; 36)	χ^2^ = 14.74 df = 2 *p* < 0.001
Monthly or less	68.8 (64.2–73.5; 265)	49.5 (39.8–59.3; 50)	
Weekly or more often	10.9 (7.8–14.0; 42)	14.9 (7.9–21.8; 15)	

CI: confidence interval.

a Missing data *n* = 6.

b Missing data *n* = 11.

c Missing data *n* = 13.

d Missing data *n* = 2.

e Missing data *n* = 1.

f Missing data *n* = 7.

g Excludes non-drinkers.

Logistic regression analysis was performed to investigate the factors associated with contraception failure after one year of use among women who consumed alcohol. The Hosmer and Lemeshow test confirmed a good fit to the data (Chi-square = 11.293; df = 8; *p* = 0.0.186) of the main effects model (not tabulated). Women aged 25–29 years (odds ratio = 2.03; 95% CI of odds 1.02–4.02; *p* = 0.044) women of Māori or Pacific ethnicity (odds ratio = 2.05; 95% CI of odds 1.11–3.78; *p* = 0.022) had nearly double the odds of using a less effective contraception in comparison with women aged 30–35 years and NZ European women respectively. Women who had no tertiary education also had an increase in the odds (odds ratio = 1.75; 95% CI of odds 0.99–3.058; *p* = 0.052) of using less effective contraception than women with some or completed tertiary education. Interestingly, women who binged *monthly or less* had lower odds (odds ratio = 0.40; 95% CI of odds 0.23–0.70; *p* = 0.001) of using a less effective contraception in comparison with women who *Never* binged. Nevertheless, there was no statistically significant difference between women who *Never* binged and those who binged *Weekly or more often* (odds ratio = 0.81; 95% CI of odds 0.34–1.89; *p* = 0.622) in using less effective contraception. There were no statistically significant differences in using less effective contraception among smokers and non-smokers, employment status, income status, and marital status (*p* > 0.05; data not shown). When the interaction of age and ethnicity was added to the model, ethnicity and age were no longer significant; however, the interaction of ethnicity with age for women aged 18–24 and those of Māori/Pacific ethnicity was significant, albeit, with a wide confidence interval of the odds ratio (odds ratio = 5.99; 95% CI of odds 1.15–31.2; *p* = 0.033; Table 4) indicating that younger Māori/Pacific drinking women were more likely to use less effective contraception. The findings for binge drinking and education were similar to the main effects model (Table 4).

**Table 4.** Factors associated with probability of contraception failure after 1 year of use among drinkers (*n* = 465).^a,b^

	Odds ratio (95% CI of odds)	*p* value
Ethnicity		
NZ European/other^c^		
Māori/Pacific	0.62 (0.15–2.64)	0.518
Asian	0.77 (0.14–4.3)	0.773
Age in years		
30–35^c^		
18–24	0.77 (0.35–1.71)	0.52
25–29	1.44(0.66–3.14)	0.36
Highest level of education		
Some or completed tertiary education^c^		
No tertiary education	1.75 (0.99–3.08)	0.055
Age *ethnicity		
18–24 years and Māori/Pacific	5.99 (1.15–31.2)	0.033
Six or more standard drinks on one occasion at least monthly or less		
Never^c^		
Monthly or less	0.38 (0.21–0.67)	**<** 0.001
Weekly or more often	0.75 (0.31–1.80)	0.517

CI: confidence incidence.

a Binary logistic regression.

b Missing = 21.

c Reference category.

* Interaction.

## Discussion

The current study aimed to describe contraception use and alcohol consumption in sexually active non-pregnant women aged 18–35 years and investigated the factors associated with contraception failure. The study focussed on women of peak childbearing years, the majority of whom were consumers of alcohol, drinking more than 2 standard drinks on a typical drinking day and six or more standard drinks on a typical drinking occasion at least ‘monthly or less’ (Table 1). Overall, 20% of drinking women (Table 2), either did not use contraception or used a method with a high failure rate, increasing the risk of an alcohol-exposed pregnancy.

Very few studies in NZ describe contraception use in women of childbearing age. A recent publication of the 2014/2015 New Zealand health survey indicated that a higher proportion of women in the lower childbearing years were using contraception in comparison with older women (88% vs 75%).^39^ Although such comparisons were not possible in our study, as it only included women in the peak childbearing years, a similar trend was observed with 95% using some form of contraception (Table 2). The NZ health survey also reported that the ‘pill’ was the most commonly used method among young women.^39^ Similarly, overseas studies have reported the pill as the most popular and consistent choice of contraception for women over time.^40,41^ In the NZ Health survey, most European women (85%) met their contraceptive needs and used modern contraceptive methods in comparison with Asian, Māori and Pacific women,^39^ similar to our findings, Māori or Pacific women are more likely to use less effective contraceptive methods (Table 3).

Studies have reported data on alcohol consumption patterns and contraception use among women of childbearing age are scarce; however, there is some evidence for unwanted pregnancies to be strongly associated with alcohol, illicit drug use, and cigarette smoking.^42,43^ A systematic review reported a lower prevalence of contraception use among women with opioid and substance use (56% vs 81%) with low proportions using effective contraception methods.^44^ Globally, alcohol consumption continues to increase in women of childbearing age and during pregnancy.^45^ In the current study, the majority consumed alcohol, with no difference in the frequency of alcohol consumption and typical day alcohol consumption according to effectiveness of contraception use (Table 3); however, statistically significant differences were found with respect to the frequency of binge drinking and use of less effective contraception, with those binging ‘monthly or less’ more likely to use effective contraception methods (*p* < 0.001; Table 3) in the univariate analysis. Nevertheless, there was no difference between women who binged ‘weekly or more often’ with those who never binged with respect to using less effective contraception (*p* = 0.51; Table 4).

In a recent global study, 25% of women who drank during pregnancy were binge drinkers,^46^ which seems to indicate a high prevalence of binge drinking women of childbearing age continue to do so in pregnancy. In a previous NZ study, 17% of women binged in early pregnancy and those most at risk were aged 16–24 years and exhibited risky drinking behaviour (women who consumed more than two standard drinks on a typical drinking day and/or binge drank at least ‘less than once a month’) prior to pregnancy.^2^ The consequences of binge drinking in pregnancy on foetal development are well established both using animal models^47,48^ and observational human studies.^49–52^

Our study did not find a significant association of less effective contraception use with other demographic characteristics except for education and an interaction between age and ethnicity. Women who did not have any tertiary education had higher odds (odds ratio = 1.75; 95% CI of odds 0.99–3.06; *p* = 0.052) of using less effective contraception (Table 4). Cannon et al.^26^ reported an association of educational level with an increased risk for an alcohol-exposed pregnancy in the United States although no clear pattern was seen. Our study also showed that alcohol-consuming women aged 18–24 years who were of Māori/Pacific ethnicity have nearly six times the odds to use less effective contraception. Despite the wide confidence interval of the odds ratio, this finding is concerning as rates of unplanned pregnancy are high among younger women and Māori and Pacific women in NZ.^53^

Studies in the United States have shown that half of the unplanned pregnancies occur in women not using contraception regularly or using ineffective methods.^54^ A simulation study estimated that 51% (42%–64%) of alcohol-exposed pregnancies could be reduced by increasing the use of effective contraception.^21^ Floyd et al.^55^ have shown a two-fold reduction in the risk for an alcohol-exposed pregnancy among ‘at risk’ women who received brief motivational counselling for both risky drinking and contraception use. Hence, understanding the use of effective contraception methods and alcohol consumption patterns among women of peak childbearing years is critical to inform policy and programmes for reducing the risk of alcohol-exposed pregnancies.

The strength of the current study is the national scope. Nevertheless, the study has several limitations. A detailed section on the limitation of the PAC has already been published.^33^ First, the somewhat lower response rate of 37%, limits the generalization of the study findings to the population. Second, respondent bias due to the self-reported nature of the study cannot be overruled. Third, the study sample was limited to women of peak childbearing age, hence, the heterogeneity in alcohol consumption patterns and contraception use across the childbearing years could not be described or accounted for in the statistical analysis. Nevertheless, the proportion of none or less effective contraception use in our sample (21%) is similar to that reported in a US study (20%),^56^ giving some confidence in the generalizability of the study findings to women of childbearing age. However, the inadequate sample size in some of the categories of interest may have also impacted the findings of the study.

Overall, the finding of the current study concurs with that of other national and international studies indicating a high prevalence of alcohol consumption and binge drinking among women of peak childbearing age. The finding that one in five alcohol-consuming women was using ineffective contraception or not using contraception is concerning. Although young Māori/Pacific women and women with lower levels of education had higher odds of an alcohol-exposed pregnancy, all women of childbearing years who regularly consume alcohol would benefit from public health efforts addressing alcohol consumption and contraception use. The increased risk of ineffective contraception among binge drinkers also cannot be overruled. Furthermore, data on contraception were captured using ‘usual’ and not ‘perfect’ use, and hence, the possibility of alcohol-exposed pregnancies due to contraception failure is likely to be higher than what we have reported.

## Conclusion

One in five women aged 18–35 years was at risk of an alcohol-exposed pregnancy. Young Māori and Pacific women and women with lower education who consume alcohol may be at higher risk for using less effective or no contraception. Public health measures to address alcohol consumption and the effective use of contraception are critical to reducing the risk of alcohol-exposed pregnancies in NZ.

**Table 5.** STROBE Checklist for Cross-sectional Studies.

		Reporting item	Page number
Title and abstract			
Title	#1a	Indicate the study’s design with a commonly used term in the title or the abstract	1
Abstract	#1b	Provide in the abstract an informative and balanced summary of what was done and what was found	2
Introduction			
Background/rationale	#2	Explain the scientific background and rationale for the investigation being reported	3
Objectives	#3	State-specific objectives, including any prespecified hypotheses	4
Methods			
Study design	#4	Present key elements of study design early in the paper	4
Setting	#5	Describe the setting, locations, and relevant dates, including periods of recruitment, exposure, follow-up, and data collection	4
Eligibility criteria	#6a	Give the eligibility criteria, and the sources and methods of selection of participants	4
	#7	Clearly define all outcomes, exposures, predictors, potential confounders, and effect modifiers. Give diagnostic criteria, if applicable	4–6
Data sources/measurement	#8	For each variable of interest give sources of data and details of methods of assessment (measurement). Describe comparability of assessment methods if there is more than one group. Give information separately for exposed and unexposed groups if applicable	4–6
Bias	#9	Describe any efforts to address potential sources of bias	n/a
Study size	#10	Explain how the study size was arrived at	4
Quantitative variables	#11	Explain how quantitative variables were handled in the analyses. If applicable, describe which groupings were chosen, and why	4-6
Statistical methods	#12a	Describe all statistical methods, including those used to control for confounding	4-6
Statistical methods	#12b	Describe any methods used to examine subgroups and interactions	4-6
Statistical methods	#12c	Explain how missing data were addressed	6
Statistical methods	#12d	If applicable, describe analytical methods taking account of sampling strategy	n/a
Statistical methods	#12e	Describe any sensitivity analyses	n/a
Results			
Participants	#13a	Report numbers of individuals at each stage of study – e.g. numbers potentially eligible, examined for eligibility, confirmed eligible, included in the study, completing follow-up, and analysed. Give information separately for exposed and unexposed groups if applicable.	Page 4
Participants	#13b	Give reasons for non-participation at each stage	n/a
Participants	#13c	Consider use of a flow diagram	n/a
Descriptive data	#14a	Give characteristics of study participants (e.g. demographic, clinical, social) and information on exposures and potential confounders. Give information separately for exposed and unexposed groups if applicable.	6
Descriptive data	#14b	Indicate number of participants with missing data for each variable of interest	Tables 1–4
Outcome data	#15	Report numbers of outcome events or summary measures. Give information separately for exposed and unexposed groups if applicable.	n/a
Main results	#16a	Give unadjusted estimates and, if applicable, confounder-adjusted estimates and their precision (e.g. 95% confidence interval). Make clear which confounders were adjusted for and why they were included	6–7
Main results	#16b	Report category boundaries when continuous variables were categorized	n/a
Main results	#16c	If relevant, consider translating estimates of relative risk into absolute risk for a meaningful time period	n/a
Other analyses	#17	Report other analyses done – e.g. analyses of subgroups and interactions, and sensitivity analyses	n/a
Discussion			
Key results	#18	Summarize key results with reference to study objectives	7
Limitations	#19	Discuss limitations of the study, taking into account sources of potential bias or imprecision. Discuss both direction and magnitude of any potential bias	9
Interpretation	#20	Give a cautious overall interpretation considering objectives, limitations, multiplicity of analyses, results from similar studies, and other relevant evidence	9,10
generalizability	#21	Discuss the generalizability (external validity) of the study results	9
Other information			
Funding	#22	Give the source of funding and the role of the funders for the present study and, if applicable, for the original study on which the present article is based	10

## Supplemental Material

sj-docx-1-whe-10.1177_17455057231161479 – Supplemental material for A cross-sectional study on alcohol and contraception use among sexually active women of childbearing age: Implications for preventing alcohol-exposed pregnancies Click here for additional data file. Supplemental material, sj-docx-1-whe-10.1177_17455057231161479 for A cross-sectional study on alcohol and contraception use among sexually active women of childbearing age: Implications for preventing alcohol-exposed pregnancies by Sherly Parackal, Mathew Parackal and Sumera Saeed Akhtar in Women’s Health

## Declarations

**Ethics approval and consent to participate:** The study received ethics approval from the University of Otago Human Ethics Committee on 25 November 2015 (Ref 15/154). Participation in the study via filling out the paper or web version of the survey implied consent to participate.

**Consent for publication:** The authors have the consent of the funders and participants to publish the findings of the study

**Availability of data and materials:** Data is not publicly available
